# Behavioral alterations are associated with vitamin B_12_ deficiency in the transcobalamin receptor/*CD320* KO mouse

**DOI:** 10.1371/journal.pone.0177156

**Published:** 2017-05-18

**Authors:** Kaveri Arora, Jeffrey M. Sequeira, Alejandro I. Hernández, Juan M. Alarcon, Edward V. Quadros

**Affiliations:** 1 Graduate Program in Molecular and Cellular Biology, SUNY Downstate Medical Center, Brooklyn, New York, United States of America; 2 Department of Medicine, SUNY Downstate Medical Center, Brooklyn, New York, United States of America; 3 Department of Pathology, The Robert F. Furchgott Center for Neural and Behavioral Science, SUNY Downstate Medical Center, Brooklyn, New York, United States of America; University of Florida, UNITED STATES

## Abstract

Vitamin B_12_ (cobalamin) deficiency is prevalent worldwide and causes megaloblastic anemia and neurologic deficits. While the anemia can be treated, the neurologic deficits can become refractive to treatment as the disease progresses. Therefore, timely intervention is critical for a favorable outcome. Moreover, the metabolic basis for the neuro-pathologic changes and the role of cobalamin deficiency in the pathology still remains unexplained. Using a transcobalamin receptor / *CD320* knockout mouse that lacks the receptor for cellular uptake of transcobalamin bound cobalamin, we aimed to determine whether cobalamin deficiency in the central nervous system produced functional neurologic deficits in the mouse that would parallel those observed in humans. Our behavioral analyses indicate elevated anxiety and deficits in learning, memory and set-shifting of a spatial memory task in the KO mouse. Consistent with the behavioral deficits, the knockout mouse shows impaired expression of the early phase of hippocampal long-term potentiation along with reduced expression of GluR1, decreased brain mass and a significant reduction in the size of nuclei of the hippocampal pyramidal neurons. Our study suggests that the *CD320* knockout mouse develops behavioral deficits associated with cobalamin deficiency and therefore could provide a model to understand the metabolic and genetic basis of neuro-pathologic changes due to cobalamin deficiency.

## Introduction

Vitamin B_12_ (cobalamin, Cbl) is an important vitamin cofactor essential for cytosolic and mitochondrial metabolic reactions [1,2]. Cbl deficiency is a health concern even today with nearly 62% pregnant women, 25 to 86% children, 21 to 41% adolescents and 11 to 90% of those over the age of 65 years, found to be Cbl deficient or have subnormal Cbl status with vegans reported to be Cbl deficient at higher rates than vegetarians [3]. Cbl deficiency is a common cause of megaloblastic anemia [4]. Cbl deficiency causes various neurologic disorders such as dementia and cognitive impairment, which adversely affect learning and memory [5,6] and, produces anxiety [7,8]. Interestingly, hematologic and neurologic symptoms do not always appear to be comorbid because in some studies, the hematologic or neurologic symptoms were present in most of Cbl deficient patients but not necessarily coexisting in the same patient [9]. While the etiology of megaloblastic anemia is well known [4], the cause of neurologic abnormalities due to Cbl deficiency is still unclear [10].

To understand the etiology of the neurologic disorder associated with Cbl deficiency, we investigated a mutant mouse in which the transcobalamin receptor (TCblR) gene (*CD320*) was ablated [10]. The TCblR/*CD320* is required for the uptake of transcobalamin (TC) bound Cbl into cells [11,12]. The TCblR/*CD320 knockout* (KO) mouse has been shown to exhibit Cbl deficiency in the central nervous system (CNS) [10], thus providing a useful model to investigate Cbl deficiency-associated disorders of the CNS.

Cbl deficiency affects two metabolic reactions where Cbl is required: First, a cytosolic reaction by the enzyme methionine synthase that requires methyl cobalamin as a cofactor to convert N^5^-methyl-tetrahydrofolate to tetrahydrofolate (THF) and homocysteine (HCY) to methionine [2]. Crucially, THF is required for the synthesis of purines and pyrimidines and its deficiency causes megaloblastic anemia [4]. The second reaction occurs in the mitochondria, where 5’-deoxyadenosyl cobalamin is a cofactor of the enzyme methylmalonyl-CoA mutase that converts methylmalonyl-CoA to succinyl-CoA [1]. Therefore, deficiency of Cbl in humans causes elevated levels of HCY and methylmalonic acid (MMA) [13,14]. Accordingly, the TCblR/*CD320* KO mouse shows elevated levels of HCY and MMA [10]. We hypothesized that alteration of these metabolic pathways in the TCblR/*CD320* KO mouse, will produce Cbl deficiency and yield abnormal behavioral performance.

We tested the TCblR/*CD320* KO mouse in behavioral tasks to assess anxiety, learning and memory, which are known to be impaired in humans with Cbl deficiency [6–8]. We evaluated these mice for anxiety disorder as well as deficits in learning of a place avoidance task and behavioral flexibility. We also analyzed synaptic physiology in the hippocampus and expression of synaptic receptors to identify potential mechanisms leading to functional deficits.

## Materials and methods

Wild type (WT) C57BL/6J mouse and the TCblR/*CD320* knockout (KO) mouse generated using CC0426/129Ola ES cells [10,12] were used in this study. All procedures were performed in compliance with the Institutional Animal Care and Use Committee of the State University of New York, Downstate Medical Center, which specifically approved this study. Groups of WT and KO (males and females, 5 months old crossbred on a C57BL/6J background) were used in various experiments in this study. The groups were as follows: One control group of wild type mice, dubbed **WT**, was fed a normal diet *ad libitum* (Picolab^®^ rodent diet 20), which contains 50 μg of vitamin B_12_ and 3 mg of folic acid per kilogram (LabDiet, St. Louis, MO, USA). A second group of wild type mice, dubbed **WT-Cbl**, was fed a Cbl deficient diet, which consisted of a modified vitamin B_12_ and folate deficient L-Amino Acid Rodent Diet with 1% Succinyl Sulfathiazole supplemented with 4 mg/kg of Folic acid and Dyetrose to enable pelleting (Dyets Inc., PA, USA). One group of TCblR/*CD320* knockout mice, dubbed **KO**, was fed a normal diet (50 μg B12, 3 mg folic acid per Kg). And, a second group of TCblR/*CD320* knockout mice, dubbed **KO+F**, received normal diet supplemented *ad libitum* with 0.5 mg/ml of folic acid in water. These groups were evaluated for various learning and cognitive behavioral deficits using the tests described below.

### Open field test

**Fig 1:**
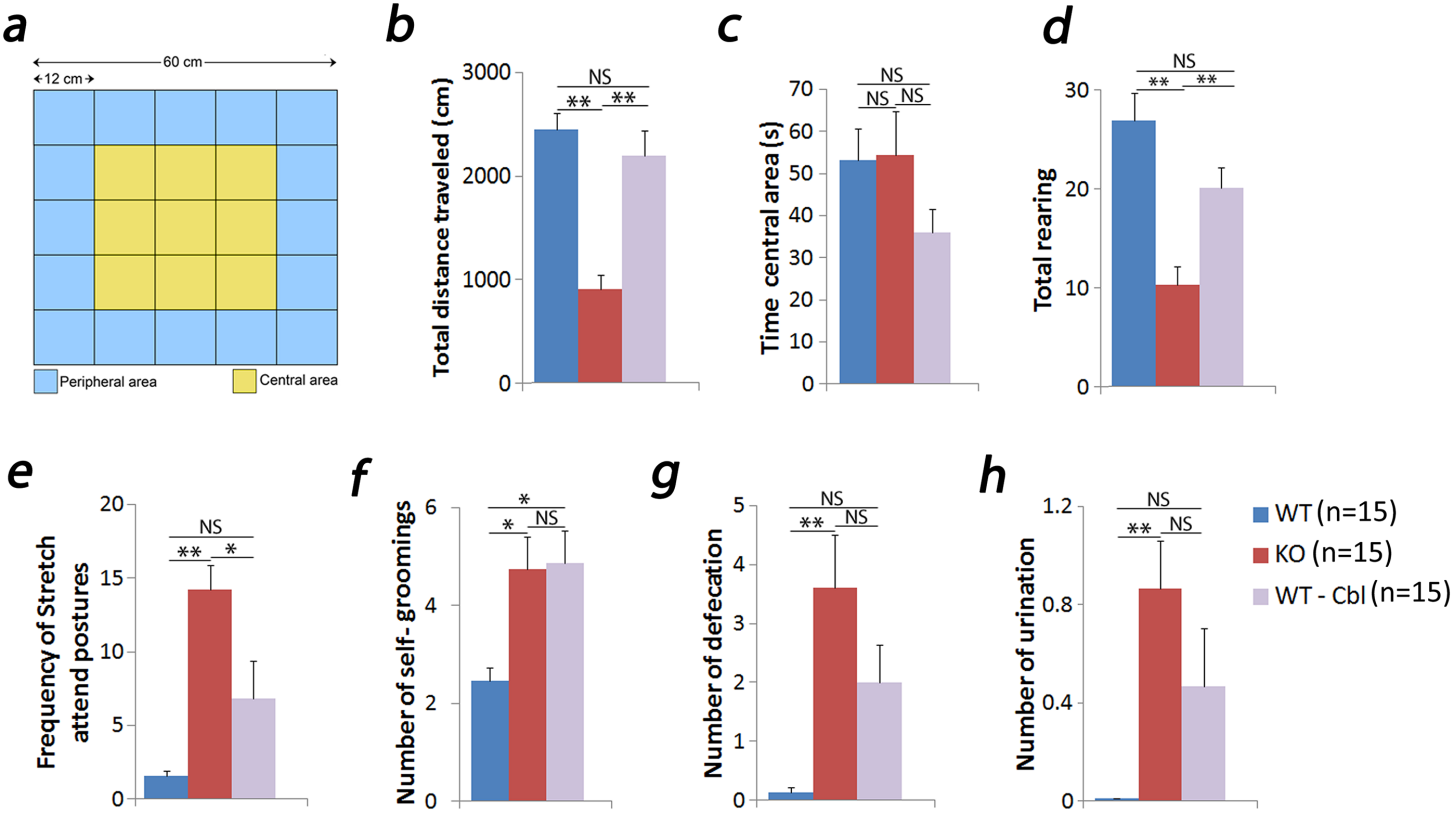
The open field test. The test arena showing central and outer zones (a). The KO mouse (n = 15) showed a decrease in total distance traveled (b), number of rearing (d) and an increase in frequency of stretch attend postures (e), number of self-grooming (f) that suggests an increase in anxiety level. This is further supported by an increase in defecation (g) and urination (h). WT on Cbl deficient diet (n = 15) showed similar trends as KO. However, it was not significantly different from WT (n = 15) (a-e, g-h). No differences were found in the time spent in central area between any of the groups (c). Results show mean ± SEM values. * p<0.05, **p<0.01, NS: not significant.

This test was used to measure anxiety, exploratory behavior and locomotor activity [15–17]. Mice were placed in the center of a box (60 x 60 cm with 40 cm wall made of white plywood and with a Plexiglass cover). Black lines divided the floor into 25 quadrants (12 x 12 cm each). The central nine quadrants were regarded as central area and the sixteen outer squares were considered peripheral area (Fig 1a). Each mouse was allowed to explore the arena for 5 minutes and the floor was cleaned with 30% ethyl alcohol and dried after each 5 minute session. All the sessions were recorded in an IBM PC computer with a camera setup 86 cm above the arena. Visual examination of the recorded sessions allowed quantification of the behavioral parameters by a person blinded to the identity of the mice. The behavioral measurements analyzed were: total distance traveled (cm), time spent in central area (s), rearing (when a mouse stands on its hind legs), frequency of stretch attend postures (where a mouse elongated its head and shoulders and then retracts to its original position), self-groomings (when a mouse scratches and licks itself while being stationery), number of defecations (fecal boli produced) and, urinations (streaks/puddles of urine). These measures were used to determine levels of anxiety in the KO mouse [18,19].

### Marble burying test

**Fig 2:**
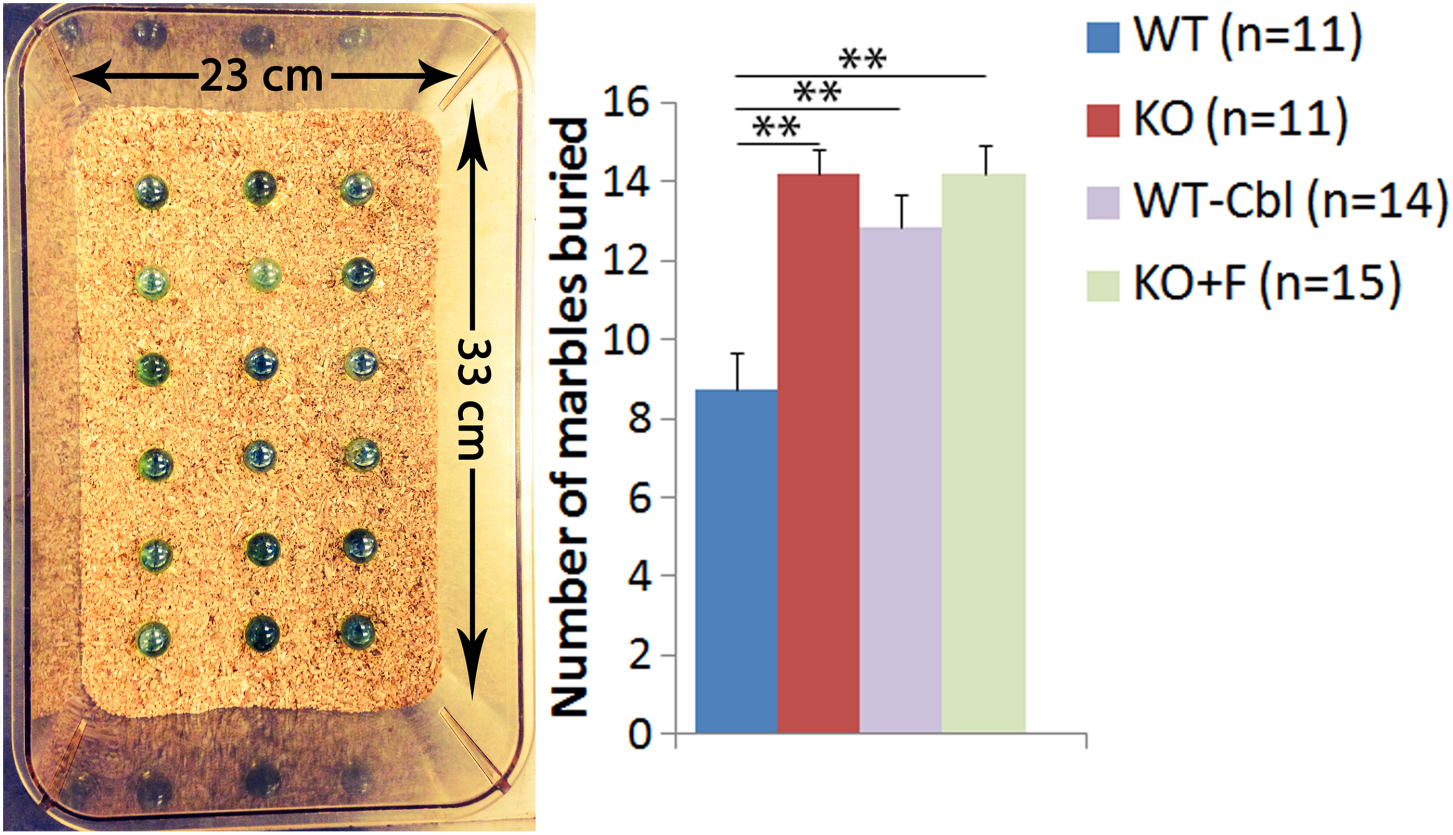
The marble-burying test. Cbl deficient groups (KO: n = 11, KO+F: n = 15 and, WT-Cbl: n = 14) buried more marbles compared to WT control group (n = 11, **p<0.01). An increase in defensive burying by Cbl deficient mice demonstrates higher anxiety level. Results show mean ± SEM values.

This test was used to investigate anxiety [20] and/or fear [21–23]. Mice were placed in a clear polycarbonate box (23 x 33 x 13 cm) filled with 5 cm of flattened sawdust bedding with eighteen glass marbles evenly spaced on the surface of the bedding in 3 columns (Fig 2). Each mouse was placed in the box for 30 minutes. The bedding was flattened and the marbles were rinsed in 50% ethyl alcohol and dried after each 30 minute session. Mice naturally tend to bury the marbles (novel object) [20–23] and the number of marbles buried to 2/3^rd^ their depth by each mouse after each session, were counted.

### Place avoidance tests

**Fig 3:**
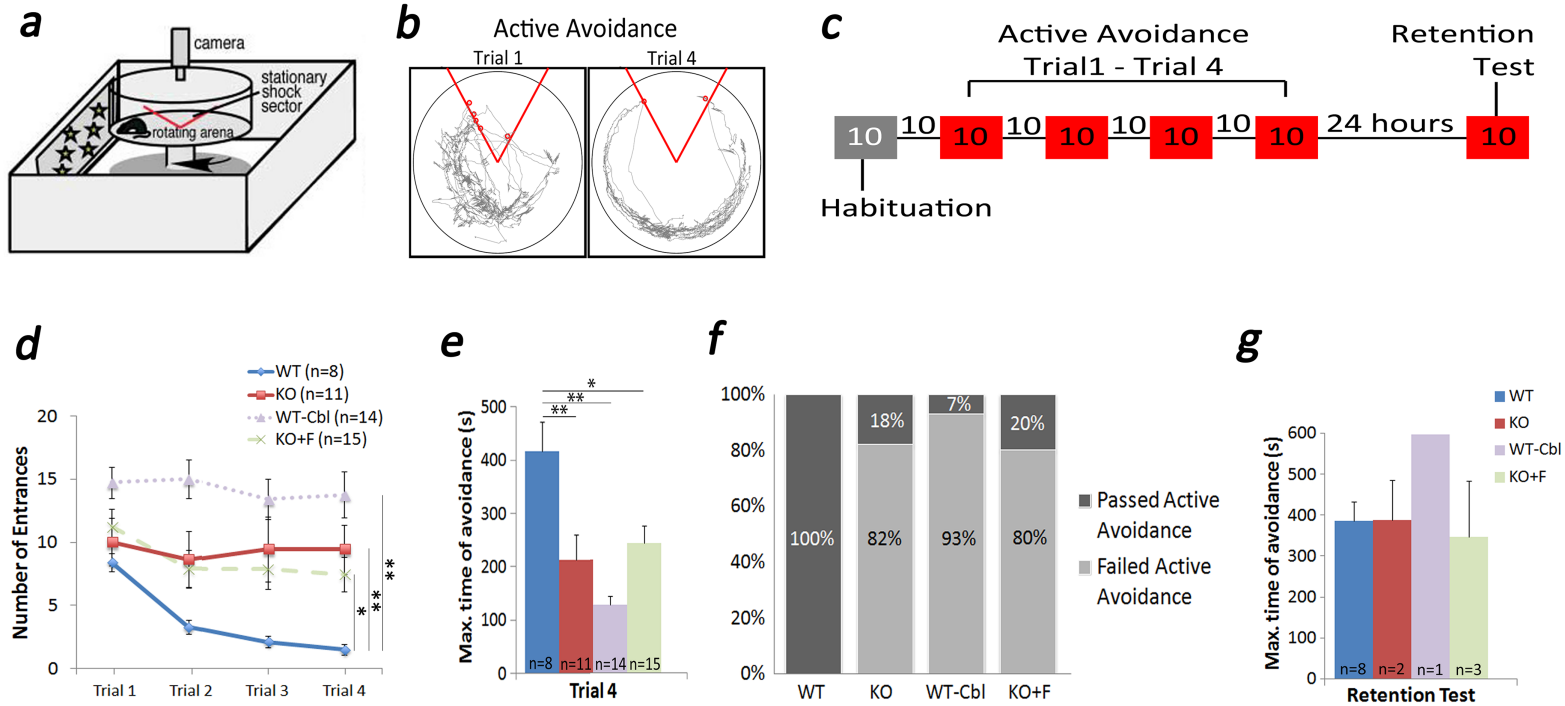
The place avoidance test for learning, memory and cognitive functions. KO mouse was unable to successfully pass the spatial learning and memory retention task using the active place avoidance paradigm. In this task, the mouse has to learn to avoid a stationary shock zone within a rotating arena using visual cues outside the arena (a). The mouse is pre-trained/habituated in the arena without a shock zone followed by 4 trials of avoidance task of 10 minutes each with an active shock zone followed by a 10 minute rest period (b and c). Learning is defined as decrease in entrances and an increase in time of avoidance with each subsequent trial. Our results showed that the KO (n = 11) mouse was unable to reduce the number of entrances into the shock zone by trial 4 compared to WT (n = 8) (d). Therefore, the maximum time of avoidance was also lower for this group in trial 4 (e). Number of entrances of 3 or less was considered as successfully completing the task. Based on this, number of KO mice passing the task was low (18%, n = 2) compared to WTs (100%, n = 8) (g). Mice that passed the test were further tested for memory retention the next day. No significant differences were found in the maximum time of avoidance between WT (n = 8) and KO (n = 2) that passed the active place avoidance test (f). We found similar results with the WT on Cbl deficient and KO with high folate diet (g). Results show mean ± SEM values. * p<0.05, **p<0.01.

An active place avoidance test was used to examine spatial learning and memory [24–27]. The task is dependent on hippocampal activity and LTP expression [28–34]. Mice were placed on a rotating stainless steel platform (rotating at 1 rpm) located in the center of a room containing cues on the walls (Fig 3a). The arena has an invisible stationary shock zone relative to the room cues (Fig 3b). The shock zone delivers an aversive foot shock of 0.2 mA at 60 Hz for 500 ms every 1.5 s until the mouse leaves the area. Mice are required to learn to avoid the shock zone by using the available spatial (room) cues. The test was conducted over two consecutive days: On day 1, mice were habituated to the (rotating) arena for 10 minutes with the shock zone off. After ten minutes of rest period, each mouse underwent four 10-minute trials of active place avoidance (shock zone ON) with 10 minutes of inter-trial breaks (Fig 3c). Behavioral measurements were recorded using an automated computer acquisition system (Bio-Signal Group Corp., New York, USA). We analyzed the number of entrances into the shock zone and the maximum time of avoidance. The decrease in number of entrances is considered to evaluate the progression of learning the task [35] and the maximum time of avoidance indicates memory retention [36], which was measured during the last trial.

### Assessment of long-term active place avoidance memory

A criterion to move forward to long-term memory testing was set for mice that only had 3 or fewer entrances into the shock zone in their fourth trial. Mice that passed this criterion were tested for long-term memory after 24 hours (on day 2). The memory retention test consisted of a 10 minute session with the shock zone off.

### Assessment of behavioral flexibility

**Fig 4:**
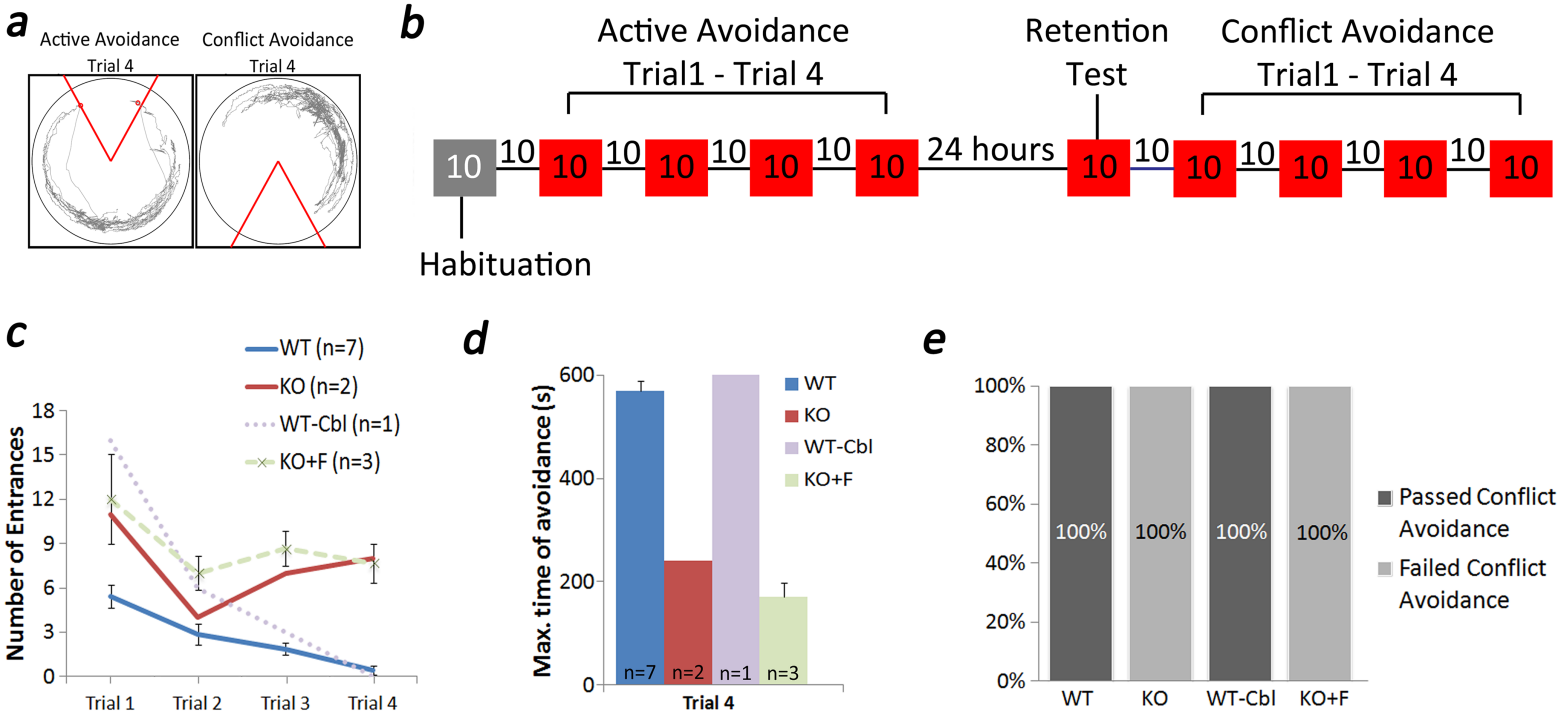
KO mice that passed active place avoidance failed the conflict place avoidance task. Conflict place avoidance task was done to identify any cognitive deficits in KO mice that passed active place avoidance. In this task the shock zone is flipped 180 degrees (a) and the mouse has to learn to avoid a new shock zone. The time for each trial was same as for active avoidance, 10 minutes (b). Our results showed that the KO (with and without high folate, n = 3 and n = 2 respectively) couldn’t pass the test with a condition of 3 or less entrances by trial 4 (c). Hence, the maximum time of avoidance was lower for these mice (d). All the WT control and WT mice on Cbl deficient diet passed and the KO mice failed the conflict avoidance test (e). Results show mean ± SEM values.

After the active place avoidance retention test, mice were given a 10-minutes break and then returned to the (rotating) arena but this time the shock zone was switched 180 degrees (Fig 4a). This protocol, dubbed conflict place avoidance (Fig 4b), allows for examination of behavioral flexibility in the form of set-shifting [37]. Four 10 minutes trials were performed with a 10 minute break between each trial. Results were analyzed the same way as for the prior active place avoidance test.

### Assessment of extended active place avoidance learning

**Fig 5:**
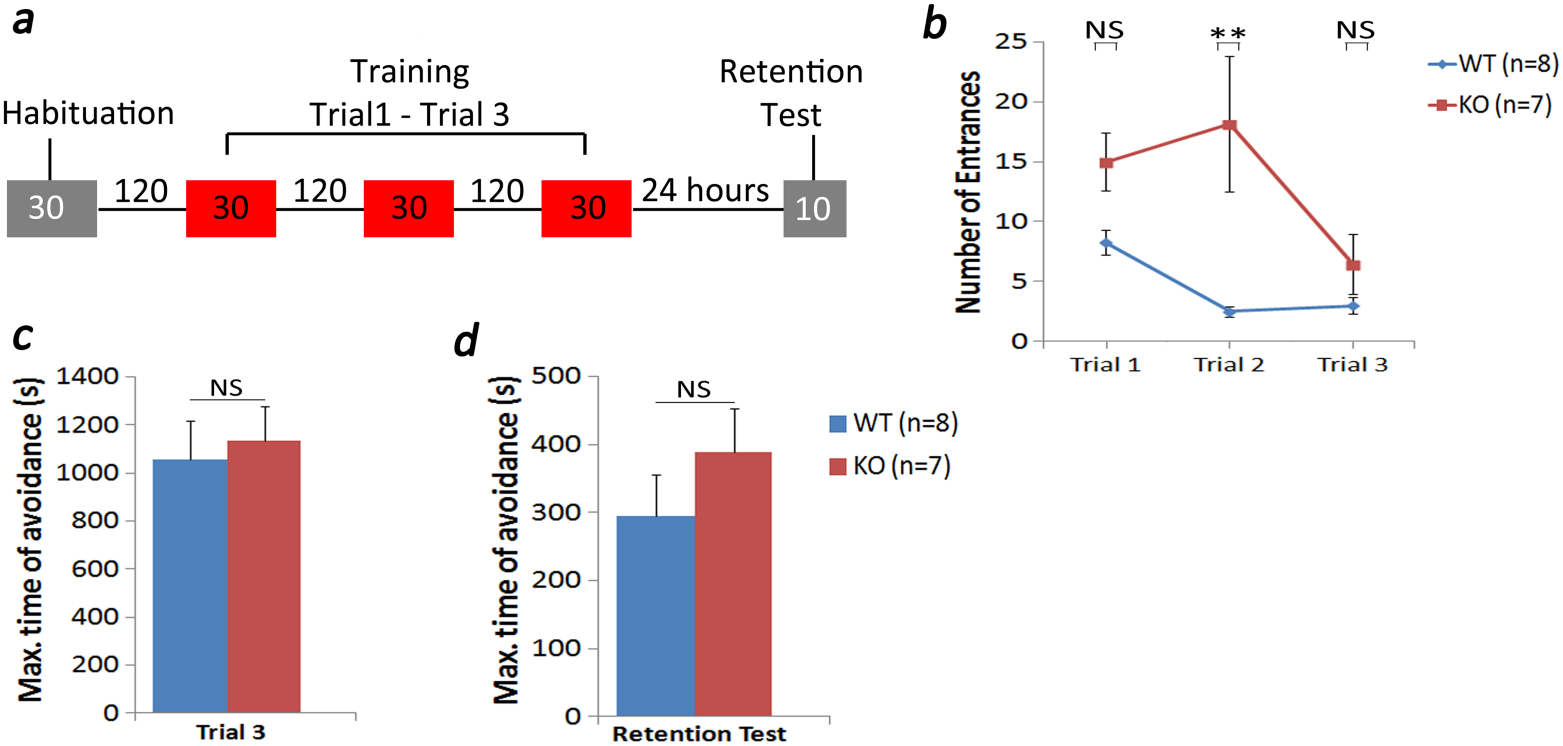
Extended learning of active place avoidance rescued learning impairment in KO mouse. For this test we extended the training trial time to 30 minutes for each trial with 120 minutes break between trials (a). A retention test for memory was conducted 24 after the last trial (trial 3). Our results showed that the KO (n = 7) mice had significantly higher number of entrances in trial 2 (**p<0.01) (b). However, the number of entrances decreased (b) and the maximum time of avoidance increased (c) by trial 3. These results were similar to what were observed in WT (n = 8). A memory retention test 24 hours after the last trial showed no difference in both groups (d). Results show mean ± SEM values. NS: not significant.

This task consisted of a 30 minutes of habituation trial followed by three active place avoidance trials of 30 minutes each with a 120-minute rest period between trials (Fig 5a). A 10-minute memory retention test was also performed 24 hr. after the last trial. The number of entrances and maximum time of avoidance were assessed as described above.

### Synaptic physiology

Mice were placed in an induction (anesthetizing) chamber for 15 minutes to allow habituation and then anesthetized with vaporized Isoflurane (5% in 100% oxygen) for 3 min. Euthanasia was performed by decapitation; brains were removed and transferred to ice cold dissection artificial cerebrospinal fluid (aCSF containing: (mM) 119 NaCl, 4.0 KCl, 7 MgSO_4_, 0.1 CaCl_2_, 26.2 NaHCO_3_, 1 NaH_2_PO_4_ and 11 Glucose saturated with 95% O_2_, 5% CO_2_). Coronal brain slices (350 μm thick) containing the right dorsal hippocampus were collected using a Vibratome 3000 and transferred to recording aCSF (aCSF containing: (mM) 119 NaCl, 4.0 KCl, 1.5 MgSO_4_, 2.5 CaCl_2_, 26.2 NaHCO_3_, 1 NaH_2_PO_4_ and 11 Glucose saturated with 95% O_2_, 5% CO_2_) and allowed to recover for 30 min at 37°C followed by 45 min at room temperature [38]. Slices were then transferred to an interface recording chamber perfused with oxygenated recording aCSF at 37°C. Schaffer-collateral to CA1 evoked field excitatory postsynaptic potentials (fEPSP) were obtained by using a pair of stimulation bipolar (FHC & Co, ME, USA) and recording (glass borosilicate pipette filled with recording aCSF 5 MΩ, Sutter Inst. Co., Novato, CA, USA) electrodes placed within the stratum radiatum of the CA1 region of hippocampus [38]. Basal synaptic transmission was assessed by generation of stimulus-response relationships between fEPSP slope amplitude responses and input voltage stimulation (0–50 V; done in duplicates) [38]. For the following synaptic plasticity evaluation, stimulation intensity was adjusted to give 40% of maximum fEPSP slope [38]. Paired-pulse facilitation (PPF), a measure of presynaptic function, was tested by delivering two closely timed pulses (10–210 ms) and assessing the percentage of facilitation of the second with respect to the first fEPSP (38). Long-term synaptic potentiation (LTP) was induced by delivering single 1-second high frequency stimulation (HFS) train at 100 Hz after 15 minutes of baseline recording (38). Tetanus envelope magnitude, a measure of depolarization during LTP induction, was determined by quantifying the initial depolarization build-up (first 50 ms) during HFS (38).

### Immunohistochemical studies

Brain collected immediately post euthanization was fixed in CARNOY’s solution (6:1:3 of ethanol: glacial acetic acid: chloroform) overnight. Fixation was followed by dehydration with increase in ethanol concentration (70%-100%), xylenes (MP Biomedicals, Santa Ana, CA) treatment and embedding in paraplast (Fisher Scientific, Hampton, NH). Coronal sections (7 μm) were cut with a Leitz microtome and mounted on silane-coated slides. Sections were cleared in xylenes and rehydrated in ethanol (100%-70%). Antigen retrieval was done for synaptophysin, PSD-95 and GluR2 staining using citrate buffer (10mM, pH 6.0 with 0.05% Tween 20) for 40 minutes at 90–100°C. Sections were washed with PBS and treated with 3% hydrogen peroxide for 5 min prior to blocking.

For synaptophysin, a rabbit polyclonal Ab (Catalog # LS-C203763; LifeSpan Biosciences Inc, Seattle, WA) was used at 1:200 dilution. Immunostaining was done using ImPress^TM^ HRP anti-rabbit IgG (Peroxidase) polymer detection kit (Catalog # MP-7401; Vector Laboratories, Burlingame, CA) according to manufacturer’s instructions.

For GluR1, a mouse monoclonal Ab (Catalog # ab174785; Abcam, Cambridge, UK) was used at 1:100 dilution. Immunostaining was done using M.O.M. Immunodetection kit (Catalog # PK-2200; Vector Laboratories, Burlingame, CA) according to manufacturer’s protocol. For synaptophysin and GluR1, counterstaining was done using haematoxylin QS (Vector Laboratories, Burlingame, CA, USA) followed by dehydration, clearing with xylenes and mounting with permount (Fisher Scientific, Hampton, NH). Imaging of the CA1 region was done using a light microscope (OLYMPUS BX51, camera DP71).

For PSD-95, a rabbit polyclonal Ab (Catalog # ab18258; Abcam, Cambridge, UK) was used at 1:100 dilution. A rabbit polyclonal Ab for GluR2 (Catalog # AB1768-I; EMD Millipore, Billerica, MA) was used at 1:200 dilution. For PSD-95 and GluR2 immunostaining, sections were incubated with normal goat serum for 1 hr at room temperature followed by primary Ab incubation for 22 hrs at 4°C. Sections were washed in PBS (2x15 min) followed by secondary Ab incubation for 16 hrs at 4°C. A goat anti-rabbit IgG H&L DyLight^®^ 550 (Catalog # ab96884; Abcam, Cambridge, UK) at 1:200 dilution was used as the secondary Ab. After incubation with secondary Ab, sections were washed in DPBS (8mM sodium phosphate, 2mM potassium phosphate, 140mM sodium chloride, 10mM potassium chloride, pH 7.4) and, counterstained with HOECHST 33342 (Catalog # 62249; ThermoFisher Scientific, Waltham, MA) using manufacturer’s protocol. Images were taken under fluorescence microscope (OLYMPUS BX51, camera DP71). Quantification of immunostaining was done using image J software.

### Anatomical studies

We examined brain weights and size of nucleus in the hippocampal pyramidal neurons of WT and KO mice. Wet weight was determined by removing and rinsing the brain in PBS and then patting it dry on kimwipes. Sections made for immunohitochemical analysis were also used for hematoxylin-eosin staining [39]. Imaging of pyramidal neurons from the CA1 region was done using a light microscope (OLYMPUS BX51, camera DP71) and the nucleus size (area as μm^2^) was measured using Image J software.

### Statistical analysis

One-way ANOVA with Tukey HSD post-hoc test was used for open-field test, marble burying test, active place avoidance, long term active place avoidance memory and, behavioral flexibility. Mann Whitney U test was used for extended active place avoidance learning task and to compare brain weights. Neuronal cell sizes were analyzed using student’s t-test. Synaptic physiology results were analyzed using two-way ANOVA with Tukey HSD post-hoc test. Immunohistochemical staining results were compared using Mann Whitney U test. Significance level was set at p<0.05.

## Results

For the characterization of the behavioral performance of TCblR/*CD320* KO (KO) mouse, in some of the studies we added two more experimental groups: (1) WT mice fed with a Cbl deficient diet (WT-Cbl) and (2) KO mice fed a folate supplement diet (KO+F). The “WT-Cbl” mice develop systemic Cbl deficiency within 3 months and the Cbl levels and metabolites in the CNS are similar to those observed in TCblR/*CD320* KO mouse (manuscript in preparation). WT-Cbl group allowed us to compare systemic Cbl deficiency induced by dietary manipulation to CNS Cbl deficiency without systemic Cbl deficiency occurring in TCblR/*CD320* KO mouse. The “KO+F” allowed us to examine the effect of high folate intake on behavioral deficits due to Cbl deficiency. The primary objective of this was to determine whether high folate intake would further deplete Cbl, worsen the CNS manifestations [40,41] and contribute to the behavioral deficits.

### Open field test

We began our examination of the TCblR/*CD320* KO mouse by determining exploratory behavior in an open field environment. The KO mouse showed significantly lower distance of total path traveled (Fig 1b); suggesting less locomotor activity in these mice. Time spent in the center area of the open field was not different between the WT, WT-Cbl and KO groups (Fig 1c). However, a decrease in rearing (Fig 1d) and increases in the number of stretch end postures (Fig 1e) and self- grooming (Fig 1f) was observed in the KO group compared to the WT group. We also measured defecation and urination as these features are also used to measure anxiety. KO mouse showed higher number of defecation (Fig 1g) and urination (Fig 1h) compared to the WT group. Our examination of the WT-Cbl mouse model revealed normal locomotor activity in this mouse (Fig 1b) and measurements of anxiogenic-associated parameters appeared to be at an intermediate range between WT and KO groups (although some differences were non-significant trends). One exception was found in the number of groomings, which was equally elevated in both KO and WT-Cbl groups (Fig 1f).

### Marble burying test

In the marble burying test, as the mice explore the environment, they tend to bury the marbles by means of a shoveling movement with their heads and vigorous treading like movements with forepaws. This action represents a defensive behavior, and when it is exacerbated, it suggests a measure of increased anxiety and/or fear. We found that the KO groups buried more marbles compared to WT mice (Fig 2). Similar results were observed for the WT-Cbl group (Fig 2). These findings are consistent with our open field findings that demonstrate anxiogenic behavior in the KO mouse.

### Active place avoidance learning and memory

Humans with Cbl deficiency show cognitive impairment, psychosis and paranoia. To characterize the performance of the KO mouse in cognitive tasks, we utilized the active place avoidance test for spatial learning and memory evaluation. In this spatial task, mice learn to avoid a specific area (shock zone) on a rotating platform to avert receiving a mild foot shock (Fig 3a). Our data indicate that avoidance learning, measured as number of entrances into the shock zone, is significantly impaired in the KO groups (KO and KO+F) and the WT-Cbl group (Fig 3d) compared to WT (control) group. Similarly, memory retention in trial 4, measured as maximum time of avoidance, is significantly decreased in KO groups as well as WT-Cbl group.

Assuming that learning is required for this task to build a memory, we considered 3 or less entrances in the last trial as an indicator of successfully completing the learning task (see Methods for details). Accordingly, we observed that 82% of KO mice, 80% of KO mice on folate diet (KO+F), and 93% of WT mice on Cbl deficient diet (WT-Cbl) showed impaired learning (Fig 3e). We next tested the retention of the place avoidance memory, measured as the maximum time of avoidance, 24 hours after the last training trial. This was tested only in mice that completed the learning test successfully. Mice that were able to learn the previous task showed intact memory from the retention test (Fig 3g).

### Behavioral flexibility test

We further investigated potential cognitive performance abnormalities in the KO mouse by characterizing their ability for behavioral flexibility. This was done by testing place avoidance trained mice on their ability to learn to avoid a new shock zone, a learning task known as conflict place avoidance [37]. Successful completion of the conflict place avoidance task requires mice to learn the new location of the shock zone without the interference by the memory of the old shock zone, a process known as cognitive control [37]. For this task, we examined only mice from each group that exhibited good memory performance (see above). As shown in Fig 3e, 82% of KO mice, 80% of KO+F mice, and 93% of WT- Cbl mice failed the active place avoidance learning task. Thus, we tested behavioral flexibility in two KO mice, 3 KO+F mice, one WT-Cbl mouse and in all eight WT control mice that were trained. Our results showed that all mice of the KO group failed to learn the conflict place avoidance task (Fig 4). Conversely, the one mouse of the WT-Cbl group successfully completed the conflict place avoidance task (Fig 4). Altogether, the data suggest that TCblR/*CD320* KO mouse exhibit a range of abnormalities in cognitive function that span from deficits in learning to impairments in behavioral flexibility, and that they can be observed on an individual basis.

### Extended learning session ameliorates the memory deficit in TCblR/*CD320* KO mouse

To test whether learning in KO mouse could be enhanced by over training, we extended the training trials from 10 (data in Fig 3) to 30 minutes (Fig 5a). This strategy successfully brought the performance of the KO mouse to levels similar to the WT mouse by the third training trial (Fig 5b). Similarly, maximum time of avoidance, as an indicator of memory retention in trial 3 was similar in WT and KO groups (Fig 5c). A memory retention test 24 hours after the last trial showed no difference in both groups (Fig 5d). It appears that extended training can rescue the spatial learning impairment in the KO mouse, thus yielding normal spatial memory retention.

### Synaptic physiology alterations in TCblR/*CD320* KO mouse

**Fig 6:**
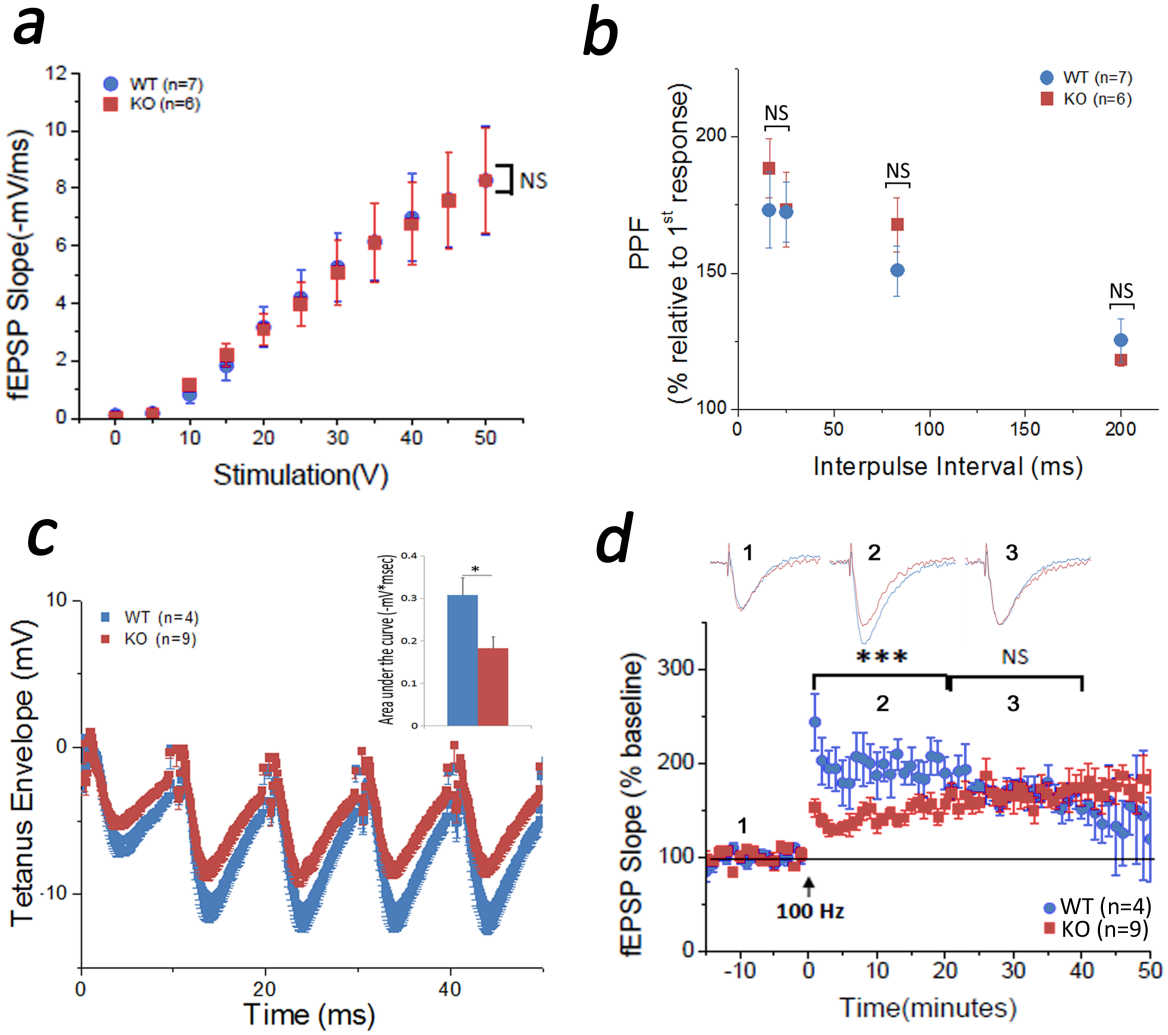
Synaptic physiology analysis. Population (extracellular) recordings at Schaffer collateral to CA1 (CA3-CA1) region showed that basal synaptic transmission was unaffected in the KO (n = 6) mouse (a). Paired pulse facilitation (PPF) was also unaffected in the KO (n = 6) from the WT (n = 7) mouse (b). However, tetanus envelope (c) and the early expression of long-term synaptic potentiation (LTP, d) were selectively affected in the KO (n = 9) compared to the WT (n = 4) mouse (*p<0.05, ***p<0.001). NS: not significant.

In order to gain some insight on the behavioral deficits observed in the KO mouse, we evaluated synaptic physiology in the hippocampus since hippocampal activity is known to be required for spatial learning and memory formation [28–34]. Population (extracellular) recordings at Schaffer collateral to CA1 (CA3-CA1) synapses showed that basal synaptic transmission is unaffected in the KO mouse (Fig 6a). Paired pulse facilitation (PPF), which reveals changes in pre-synaptic neurotransmitter release [38,42], also showed similar profile in both WT and TCblR/*CD320* KO mouse (Fig 6b). However, the magnitude of the tetanus envelope (measured as the initial depolarization build-up during HFS), which reveals changes in post-synaptic depolarization that drives LTP induction [38], was smaller in the TCblR/*CD320* KO mouse compared to WT (Fig 6c). Concomitantly, we observed LTP deficits in the TCblR/*CD320* KO compared to the WT mouse (Fig 6d). Interestingly, the deficit affected the early, but not the late, expression of LTP (Fig 6d).

### Immunohistochemical analysis

**Fig 7:**
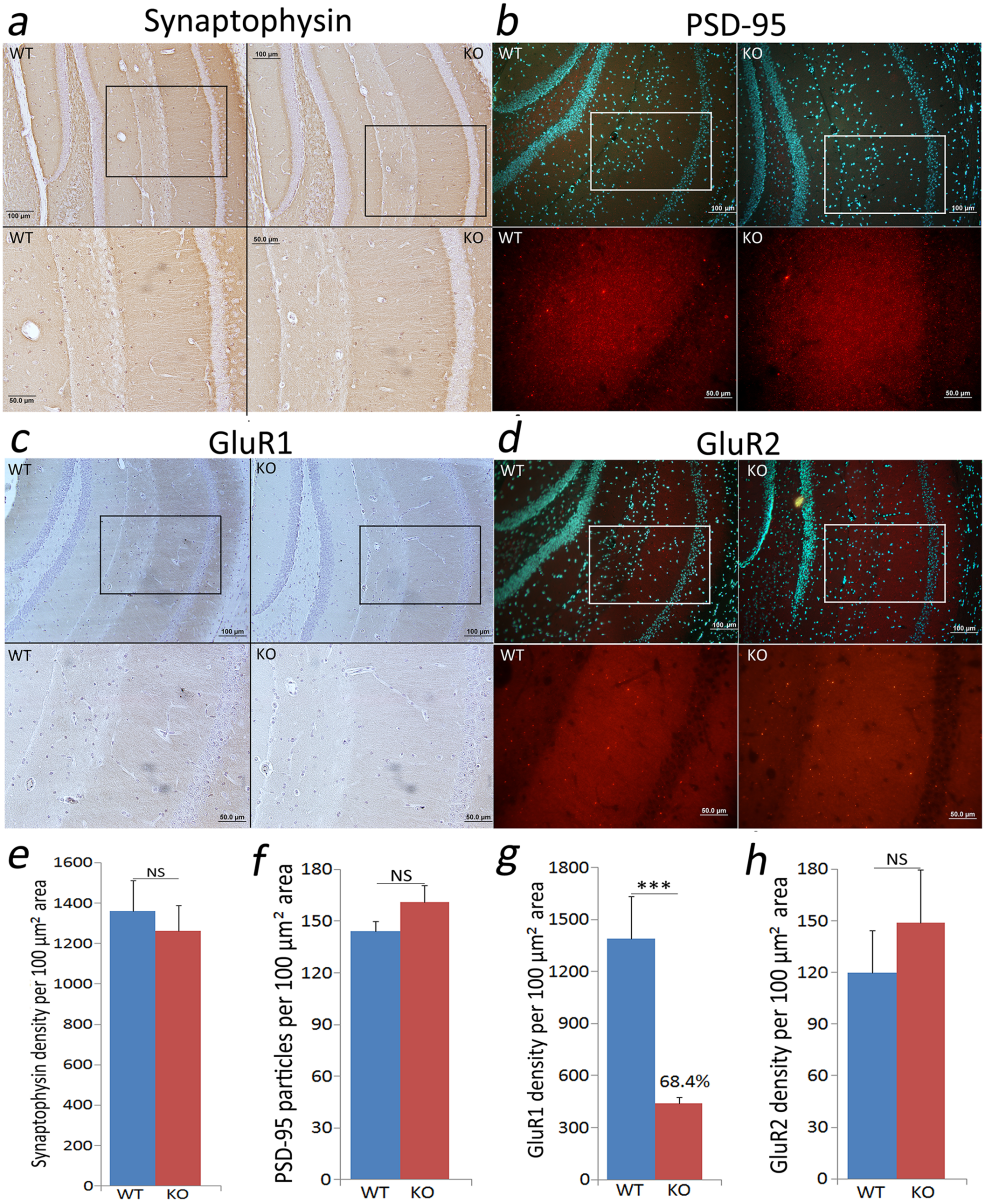
Immunohistochemical analysis in the CA1 region of hippocampus. Expression of synaptophysin where Schaffer Collateral projects to connect and form CA3-CA1 synapses. was unaltered in the TCblR/CD320 KO (n = 2) mouse (a, e). PSD-95 expression also remained the same in the KO (n = 2) as in the WT (n = 2) mouse (b, f). GluR1 expression in the KO (n = 2) mouse was significantly lower (c) with ~68% reduction in the expression level (g). GluR2 expression remained the same in the KO (n = 2) and the WT (n = 2) mouse (d, h). Panel a, b, c, d show the region of CA1 in box (image on top) magnified below (image at bottom). Although, the quantification was done for stratum radiatum region only. Panel e, f, g, h show mean ± SEM values. (***p<0.001), NS: not significant.

To further complement our evaluation of synaptic function, we studied the expression of the pre-synaptic and post-synaptic markers synaptophysin and PSD95, respectively [43], and of the AMPA receptor subunits GluR1 and GluR2, which support basal synaptic transmission as well as LTP [44,45]. The expression of each of these protein factors was studied in the CA1 region of hippocampus where Schaffer Collateral projects to connect CA3-CA1 synapses (i.e. *stratum radiatum* layer). We found no difference in the expression levels of synaptophysin (Fig 7a and 7e) or PSD-95 (Fig 7b and 7f) between WT and the TCblR/*CD320* KO mouse; suggesting no dramatic alteration at the synapse population level in the KO mouse. However, a significantly lower expression of GluR1 (Fig 7c and 7g) and no change in GluR2 expression (Fig 7d and 7h) were found in the TCblR/*CD320* KO mouse; suggesting a connection between reduced GluR1-containing AMPA receptors and the observed LTP deficit.

### Anatomical alterations in TCblR/*CD320* KO mouse

**Fig 8:**
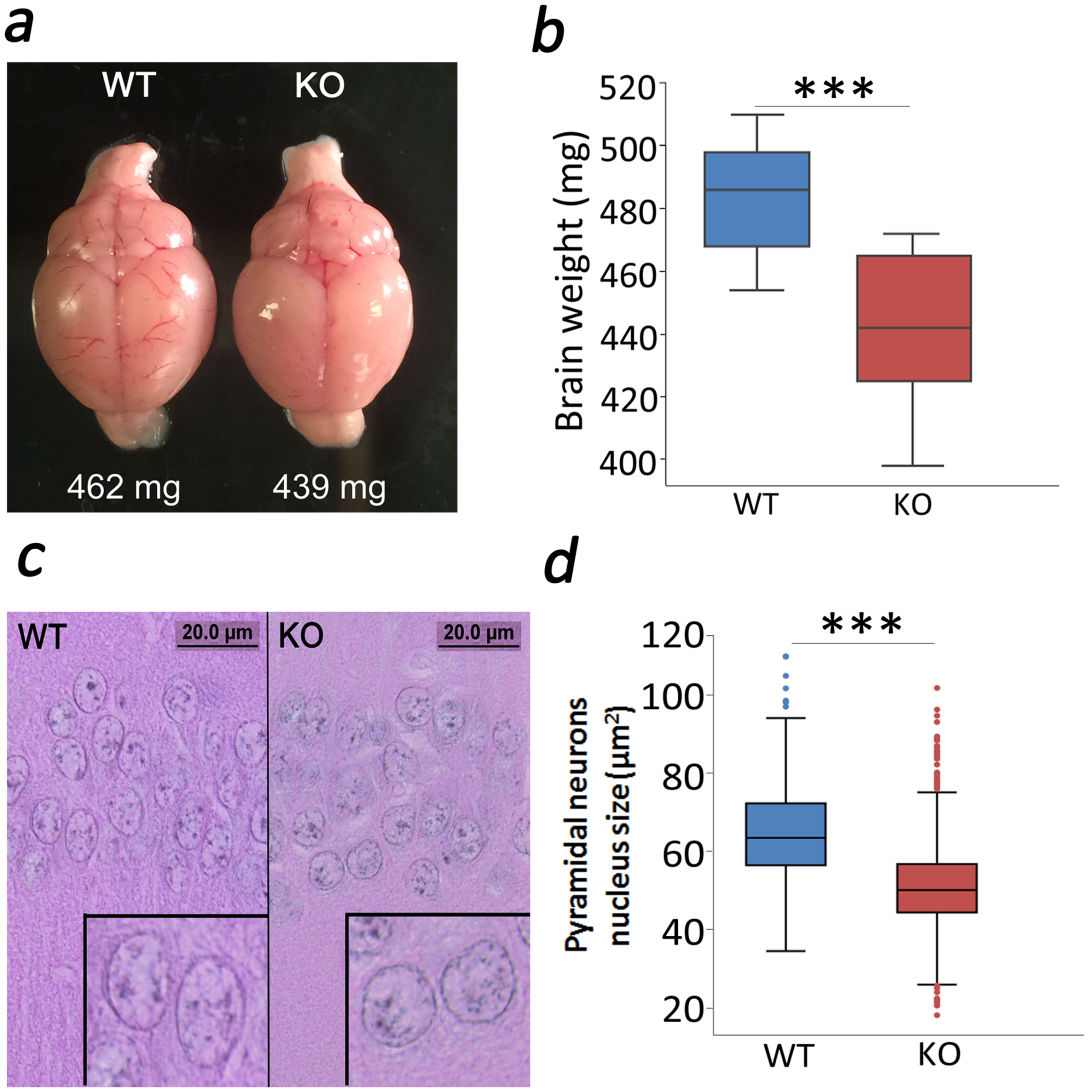
Decreased brain mass in the CD320 KO mouse. Brain mass in KO mouse (n = 18) was significantly less (***p<0.001) than in WT mouse (n = 14) (a, b). Moreover, hippocampal CA1 pyramidal cell nucleus size was also significantly lower (***p<0.001) in KO (number of cells counted = 539 from 5 mice) compared with WT (number of cells counted = 561 from 5 mice) (c, d).

We also evaluated brain mass and hippocampal neuronal nucleus size of the TCblR/*CD320* KO mouse. On average, the brain mass in the KO mouse was about 50 mg (~8%) less than WT (Fig 8a and 8b). The size of nuclei of hippocampal CA1 pyramidal cell bodies was significantly smaller in KO compared with WT mouse (Fig 8c and 8d).

## Discussion

Cbl deficiency is still a cause of health concern. In addition to megaloblastic anemia, a major functional deficit in patients with Cbl deficiency is neurologic disorder [4–6]. While megaloblastic anemia has been thoroughly studied and can be treated, the mechanisms underlying the neurologic manifestations are still unknown [10]. To understand the mechanisms underlying functional deficits occurring due to Cbl deficiency, we used a TCblR/*CD320* KO mouse. Lack of TCblR restricts Cbl uptake which causes Cbl deficiency in the CNS of the KO mouse [10,11]. In this study we observed that the KO mouse displays behavioral alterations that parallel those observed in the human conditions (i.e. anxiety disorders, learning and cognitive deficits). Further insight into the behavioral alterations was gained by studying synaptic function; we found impaired LTP, particularly the early expression of LTP, and a lower expression of the AMPA receptor subunit GluR1 in the KO mouse. The KO mouse also showed brain atrophy revealed as a decrease in neuronal cell size, potentially due to nutrient deficiency and metabolic stress contributing to the observed functional deficits. The TCblR/*CD320* KO mouse therefore, provides a suitable animal model to study the neurologic disorder associated with Cbl deficiency.

Several Cbl deficient animal models have been generated by dietary manipulation in baboons [46], the fruit bat [47], rhesus monkeys [48], and gastrectomized rats [49]. However, no extensive behavioral testing was done in these models and no clinical phenotype was observed. Deleting cubilin, the membrane receptor for the absorption of intrinsic factor-Cbl [50] and megalin, the protein involved in renal reabsorption of Cbl and potentially in fetal development were embryonic lethal [51], The non lethal outcome of deleting the CD320 gene, and selective Cbl depletion in the CNS has provided an unique model to study the neupathologic consequences of Cbl deficiency in a mouse model.

Higher anxiety is a well-reported neurologic condition associated with Cbl deficiency in humans [7,8] which is also observed in the TCblR/*CD320* KO mouse. Similarly, humans with Cbl deficiency show impaired learning and memory capabilities [5,6], which are also observed in the KO mouse as alterations in place avoidance learning and memory performance and deficits in behavioral flexibility. These findings are consistent with studies that showed cognitive deficits in rats on vitamin B-complex deficient diet [52,53]. Importantly, our observations that the KO and KO+F groups were equally impaired in learning and memory support the notion that the behavioral phenotype is only due to Cbl deficiency as supplementing the food with folate does not improve or worsen the learning impairment of the KO mouse. Notably, TCblR/*CD320* KO mice that underwent extended learning trials showed normal memory retention suggesting that the functional deficits can be restored by extensive training.

Our synaptic physiology and immunohistochemical results provided further insight into the potential mechanism underlying behavioral alterations observed in the TCblR/*CD320* KO mouse. Unchanged expression of synaptophysin, PSD-95 and basal synaptic transmission in the KO mouse suggest normal number and basal function of synapses in this mouse. Unchanged PPF ratios suggest no impairment in pre-synaptic neurotransmitter release in the KO mouse. However, reduced tetanus envelope in the KO mouse suggests deficient depolarization to induce LTP. Moreover, impaired initiation of LTP suggests a failure in the recruitment of post-synaptic mechanisms involved in the early expression of LTP.

In CA1 synapses, basal synaptic transmission is dependent on AMPA receptor function [54] whereas LTP expression requires both AMPA as well as NMDA receptors [54]. AMPA receptors are heterodimers of GluR1-4 subunits [44]. Basal synaptic transmission is supported by AMPA receptors constituted of GluR1/2 [55] and GluR2/3 [56] heterodimers. It has been observed that in absence of GluR1 (i.e. GluR1 KO mouse), GluR2/3 and/or GluR2/4 containing AMPA receptors can support normal basal synaptic transmission [55]. However, this substitution cannot support the expression of LTP [55,57]. Indeed, the early expression (induction and initiation) of LTP is directly dependent on the modulation (i.e. phosphorylation) and incorporation of GluR1-containing AMPA receptors at the post-synaptic membrane [44]. Furthermore, while GluR1-containing AMPA receptors are key for the initiation of LTP, GluR2-containing AMPA receptors are necessary for the late expression (consolidation and maintenance) of LTP [44]. Studies done in GluR1 KO mouse have shown lower LTP response in the early phase, which in the late phase corresponds to the WT mouse [57]. Lower expression of GluR1 in the TCblR/*CD320* KO mouse could explain the initial deficit in LTP expression, which is then recovered by the gradual incorporation of GluR2-containing AMPA receptors. Similarly, basal synaptic transmission in the TCblR/*CD320* KO mouse could be supported by GluR2/3 and/or GluR2/4 containing AMPA receptors. While our tetanus envelope analysis could entail a deficit in NMDA receptor function [38], the fact that we can observe LTP expression indicates recruitment of post-synaptic plasticity mechanisms despite this putative deficiency. Interestingly, the connection between reduced GluR1 and impaired initiation of LTP could be further extended to the delayed learning observed in the KO mouse, which needed more time and training than WT to acquire the place avoidance memory.

Reduction in GluR1 has also been found in neurons from a mouse model of Alzheimer’s disease (Tg2576); although, these neurons also showed lower PSD-95 levels [58]. Like the TCblR/*CD320* KO mouse, the Tg2576 mouse model shows normal basal synaptic transmission and PPF but deteriorated LTP [59]. The Tg2576 mouse model accumulates beta-amyloid and generates oxidative stress as well as mitochondrial dysfunction [60], and reduction of oxidative stress in this mouse model prevents learning and memory deficits [61]. Metabolic stress has also been associated with brain dysfunction due to elevated homocysteine (HCY) levels [52,53,62–64]. HCY is a known neurotoxin [65] that can cause oxidative stress [66,67], endoplasmic reticulum stress [68] and mitochondrial toxicity [69], contributing to synaptic dysfunction and memory deficits [70]. HCY is also known to regulate levels of NMDA and AMPA receptors [71]. As indicated above, proper functioning of these receptors is key for the expression of synaptic plasticity (e.g. LTP) as well as learning and memory [44,72,73]. Cbl deficiency causes elevated HCY levels [10,13] and produces persisting brain atrophy [63,64,74,75]. Previous studies done in vitamin B-complex deficient rats showed elevated HCY levels and structural deficits in the brain; namely reduced thickness of CA1 pyramidal layer without any noticeable gliosis [52]. These observations are consistent with our findings of smaller brain mass and size of nuclei in pyramidal neurons which correlates with cell body size in the hippocampal CA1 region [76]. In summary, Cbl deficiency in the CNS of the TCblR/*CD320* KO mouse produces anxiety and behavioral deficits. In addition, learning, memory and cognitive deficits are observed that can be rescued by extended learning period. The alterations in LTP are consistent with reduced GluR1 and compensatory mechanisms. Studying changes in expression of genes, involved in metabolic stress, in our TCblR/*CD320* KO mouse is likely to provide additional insight into the neuropathology of Cbl deficiency. Understanding these mechanisms could provide potential treatments for neurological disorders associated with Cbl deficiency.

## Supporting information

S1 Dataset Fig 1 data. (XLSX)

S2 Dataset Fig 2 data. (XLSX)

S3 Dataset Fig 3 data. (XLSX)

S4 Dataset Fig 4 data. (XLSX)

S5 Dataset Fig 5 data. (XLSX)

S6 Dataset Fig 6 data. (XLSX)

S7 Dataset Fig 7 data. (XLSX)

S8 Dataset Fig 8 data. (XLSX)
